# Efficient Energy Transfer Down-Shifting Material for Dye-Sensitized Solar Cells

**DOI:** 10.3390/ma18143213

**Published:** 2025-07-08

**Authors:** Emeka Harrison Onah, N. L. Lethole, P. Mukumba

**Affiliations:** 1 Physics Discipline, Department of Computational Sciences, University of Fort Hare, Private Bag X1314, Alice 5700, Eastern Cape, South Africa; nlethole@ufh.ac.za (N.L.L.); pmukumba@ufh.ac.za (P.M.); 2 SAMRC Microbial Water Quality Monitoring Centre, University of Fort Hare, Private Bag X1314, Alice 5700, Eastern Cape, South Africa

**Keywords:** luminescent down-shifting, dye-sensitized solar cells, UV absorption, energy transfer, solid-state technique

## Abstract

Dye-sensitized solar cells (DSSCs) are promising alternatives for power generation due to their environmental friendliness, cost effectiveness, and strong performance under diffused light. Conversely, their low spectral response in the ultraviolet (UV) region significantly obliterates their overall performance. The so-called luminescent down-shifting (LDS) presents a practical solution by converting high-energy UV photons into visible light that can be efficiently absorbed by sensitizer dyes. Herein, a conventional solid-state technique was applied for the synthesis of an LDS, europium (II)-doped barium orthosilicate (BaSiO_3_:Eu^2+^) material. The material exhibited strong UV absorption, with prominent peaks near 400 nm and within the 200–300 nm range, despite a weaker response in the visible region. The estimated optical bandgap was 3.47 eV, making it well-suited for UV absorbers. Analysis of the energy transfer mechanism from the LDS material to the N719 dye sensitizer depicted a strong spectral overlap of $2\times 10^{10}M^{-1}{cm}^{-1}{nm}^{4}$, suggesting efficient energy transfer from the donor to the acceptor. The estimated Förster distance was approximately 6.83 nm, which matches the absorption profile of the dye-sensitizer. Our findings demonstrate the potential of BaSiO_3_:Eu^2+^ as an effective LDS material for enhancing UV light absorption and improving DSSC performance through increased spectral utilization and reduced UV-induced degradation.

## 1. Introduction

Solar energy, being abundant and environmental friendly, has emerged as a key alternative clean, sustainable, and renewable energy source [1,2,3]. Among various photovoltaic technologies, dye-sensitized solar cells (DSSCs) have gained substantial interest due to their low manufacturing cost, eco-friendliness, and promising performance under diffused-light conditions [4,5,6,7,8]. Despite these advantages, DSSCs continue to have drawbacks that impair their performance, especially their limited spectral response in the ultraviolet (UV) portion of the solar spectrum.

The low UV absorption by conventional dye molecules used in DSSCs, such as N719 dyes, results in underutilization of a significant portion of incident solar energy [9,10]. Therefore, high-energy UV photons can induce photodegradation of key DSSC components, such as dyes and electrolytic materials, thereby reducing both the performance and operational stability of the cells. To address these issues, researchers have explored various spectral management approaches, with luminescent down-shifting (LDS) standing out as a promising approach [11,12,13,14].

High-energy UV photons are absorbed by LDS materials and re-emitted at longer visible wavelengths, where DSSC dyes can efficiently absorb them. The efficiency and stability of DSSCs could be increased by this photon energy conversion mechanism, which also reduces UV-induced degradation and enhances the spectrum match between incident light and the dye’s absorption profile. Rare-earth-doped inorganic phosphors have gained significant attention among various LDS candidates due to their excellent photostability and tunable emission properties [15,16,17]. While it is true that DSSCs peaked in research interest over about a decade ago, recent advances have rekindled interest due to their potential in niche applications such as indoor photovoltaics, building-integrated photovoltaics, and portable devices. The main limitation of DSSCs remains their low spectral response to UV light, which spectral converters like down-shifting materials are developed to address. Therefore, our research focuses on developing cost-effective and efficient down-shifting materials to enhance the spectral responsivity of DSSCs in the UV region.

In this regard, barium orthosilicate doped with europium (II) ions (BaSiO_3_:Eu^2+^) is an interesting material for LDS. Eu^2+^-doped phosphors are known for their broad excitation bands in the UV region and strong emission in the visible spectrum, owing to the 4f^6^5d^1^ → 4f^7^ electronic transitions of the Eu^2+^ ion [18,19,20]. BaSiO_3_ itself provides a robust host matrix that supports the stable incorporation of Eu^2+^ ions and facilitates efficient luminescence [21,22]. It is particularly suited for LDS in DSSCs due to its high UV absorbance, enabling efficient utilization of UV portion of the solar spectrum. The absorption profile of the N719 dye sensitizer matches well with the down-shifting behavior of BaSiO_3_:Eu^2+^, which exhibits a strong blue-green emission upon UV excitation. Additionally, it is easily integrated as surface or interfacial coatings due to its compatibility with DSSCs. It has a low absorption loss due to the large Stokes shift, which reduces back-absorption. Therefore, these characteristics position BaSiO_3_:Eu^2+^ as a suitable spectral converter for DSSCs.

Herein, we present the synthesis and optical and luminescent properties of the BaSiO_3_:Eu^2+^ material, evaluating its feasibility as a spectral conversion material. A conventional solid-state technique was applied for the synthesis of the material. Scanning electron microscope (SEM) and energy-dispersive X-ray spectroscopy (EDS) were employed to characterize surface morphology and elemental composition. UV–vis-diffused reflectance spectroscopy was used to estimate the optical band gap. Photoluminescent (PL) spectroscopy was utilized to analyze excitation and emission spectra and Förster theory to quantify the energy transfer efficiency through overlap integral and Förster distance estimation. Barium orthosilicate-doped europium (II) ions demonstrated a strong absorption profile within the UV range, with an estimated optical bandgap of 3.47 eV, making it suitable for UV-absorbing down-shifting material for DSSCs. The distance between the donor and acceptor molecules as well as the strength of the spectral overlap determined the efficiency of the energy transfer. The energy transfer mechanism demonstrated a strong integral overlap, i.e., $J\left(\lambda \right)$, between the LDS donor material and the N719 dye acceptor molecules, with an estimated value of $2\times 10^{10}\;M^{-1}{cm}^{-1}{nm}^{4}$. The distance between these entities was <10 nm, indicating efficient energy transfer. The novelties of the study include the presented quantitative analysis of energy transfer efficiency via Förster resonance energy transfer (FRET), including direct numerical estimation of spectral overlap integral J(λ) and the Förster distance (R_0_), which have never been reported. Therefore, by analyzing the luminescent behavior of the phosphor and spectral overlap $J\left(\lambda \right)$ with typical a DSSC N719 dye absorption profile, the study aims to assess its potential to improve the spectral responsivity and ultimately the photovoltaic performance of DSSCs.

## 2. Materials and Methods

### 2.1. Experimental Procedure

Barium carbonate (BaCO_3_), 99.99%; silicon dioxide (SiO_2_), 99.99%; and europium oxide (Eu_2_O_3_), 99.99%, all analytically graded from the Sigma-Aldrich—Merck Group, Johannesburg, South African division, were used as the starting materials during the preparation of the sample.

The europium (II)-doped barium silicate material Ba_(1−x)_SiO_3_:xEu^2+^ (x = 0.03) was prepared by the conventional solid-state method. This value of concentration was selected to avert concentration quenching. Higher doping concentration may cause non-radiative energy losses due to much ion clustering, whereas lower levels may result in insufficient luminescent intensity for an effective down-shifting process. Initially, the starting materials were weighted according to the nominal stoichiometry of Ba_0.97_SiO_3_:0.03Eu^2+^ phosphor, and then, the powders were mixed and milled thoroughly using a mortar and pestle. The chemical reaction that governed the stoichiometry calculation was

2BaCO_3_ + 2SiO_2_ + 2Eu_2_O_3_ → 2Ba_1−x_SiO_3_:xEu^2+^ + 2CO_2_ + 3O_2_↑

**Figure 1:**
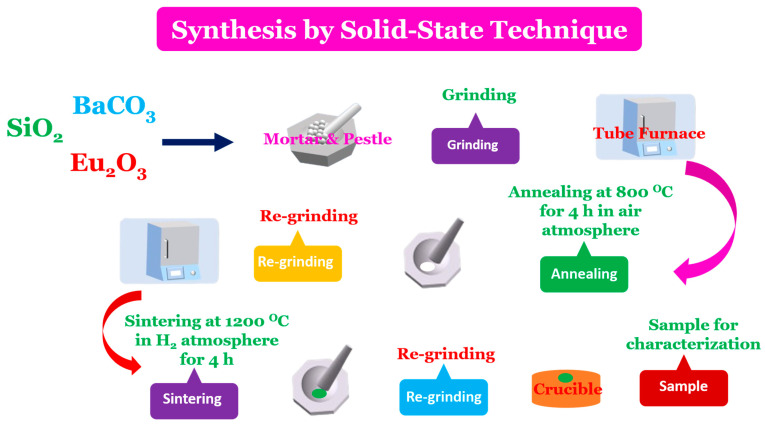
Synthesis of the BaSiO_3_:Eu^2+^ by solid-state technique.

The ground sample was placed in an alumina crucible and subsequently annealed at 800 °C for 4 h in a vacuum using a tube furnace—Chengyi instrument, Zhengzhou, China. After re-grinding, the sample was sintered to 1200 °C for 4 h under an H_2_-reducing atmosphere at approximately 100 standard cubic centimeters per minute (SCCM), in the same instrument. A reducing hydrogen atmosphere allows and favors stabilization of Eu^2+^ over Eu^3+^. The nominal compound was obtained after cooling down of the programmable furnace. The solid-state synthesis route applied is shown in Figure 1 below.

Microstructural analysis was performed to study the surface morphologies of the phosphors using the scanning electron microscope (SEM) model JSM-7800F—JOEL Limited, Tokyo, Japan with an electron beam voltage of 5.0 keV and further equipped with energy-dispersive X-ray spectroscope (EDS) for elemental identification. A UV–vis spectrophotometer (PerkinElmer LAMDA 35)—PerkinElmer Incorporated, Waltham, MA, USA was used for the analysis of diffuse reflectance, from which the absorbance spectrum and the absorption edge for band gap estimation were determined. Photoluminescent (PL) emission was performed using the Cary Eclipse Spectrophotometer—Agilent Technologies, Santa Clara, CA, USA and the quantum yield measurement was carried out using the FLS980 spectrometer—Edinburgh Instruments, Scotland, UK. All characterizations were performed at room temperature, and all plots were created using Python 3.9.

### 2.2. Optical Band Gap Estimation

The optical band gap is the minimum photon energy required to promote an electron from the valence band to the conduction band, which can be determined from the reflectance or absorbance spectra [23,24]. In this regard, we recall that photon energy (E) is related to the wavelength (λ) as represented by Equation (1).

(1) $$E=\frac{hc}{\lambda }$$

where Planck’s constant (h) = 4.136 × 10^−15^ eV.s, and the speed of light (c) = 3 × 10^8^ m/s. As $E\propto \frac{1}{\lambda }$ therefore, increasing photon energy implies decreasing the wavelength. With respect to the reflectance (R), normalization is crucial to scale the reflectance data to the [0, 1] range. This is to mitigate amplitude-based effects in derivative analysis in order to realize a unity peak of the reflectance spectrum. The normalization technique applied is depicted in Equation (2).

(2) $$R_{norm}=\frac{R-R_{min}}{R_{max}-R_{min}}$$

Computation of the first derivative of the normalized reflectance with respect to the photon energy $\left(\frac{dR}{dE}\right)$ is also crucial to highlight inflection points corresponding to rapid changes in reflectance, which indicate electronic transition from the valence band to conduction band [25,26]. Since R is a function of the wavelength, we can obtain $\left(\frac{dR}{dE}\right)$ by

(3) $$\frac{dR}{dE}=\frac{dR}{d\lambda }.\frac{d\lambda }{dE}$$

Recall the relationship between wavelength and photon energy

(4) $$\lambda =\frac{hc}{E}$$

A derivative of the wavelength with respect to photon energy yields

(5) $$\frac{d\lambda }{dE}=-\frac{hc}{E^{2}}$$

Substituting Equation (5) into Equation (3) gives

(6) $$\frac{dR}{dE}=-\frac{hc}{E^{2}}.\frac{dR}{d\lambda }$$

Band gap energy $E_{g}$ can then be estimated by the value where $\frac{dR}{dE}$ reaches its maximum, which is the energy corresponding to the maximum first derivative. This expression is shown in Equation (7). The derivative-based approach provides a computationally efficient method to estimate optical band gaps from reflectance spectra as a function of wavelength, which represents a useful tool in exploratory material analysis.

(7) $$E_{g}=argmax\left(\frac{dR}{dE}\right)$$

### 2.3. Estimation of Overlap Integral and Förster Distance

In order to enable efficient energy transfer, there must be a spectral overlap between the emission spectrum of the donor and the absorption spectrum of the acceptor [27,28,29]. The overlap integral $J\left(\lambda \right)\;(M^{-1}{cm}^{-1}{nm}^{4})$ is governed by Equation (8) below.

(8) $$J\left(\lambda \right)=\int_{0}^{\infty }F_{D}\left(\lambda \right)\epsilon_{A}(\lambda )\lambda^{4}d\lambda$$

where $F_{D}$ is the normalized emission spectrum of the donor, which is a dimensionless entity; $\epsilon_{A}$ is the molar absorption or extinction coefficient of the acceptors—a measure of how strongly chemical species absorb light at a particular wavelength in $M^{-1}{cm}^{-1}$; λ is the wavelength of the incident light in nm.

The distance between donor and acceptor molecules as well as the strength of the overlap integral determines the efficiency of the energy transfer. Therefore, the smaller the distance between the donor and the acceptor molecules and the larger the overlap integral, the greater the efficiency of the energy transfer. A convenient way to express the J-overlap integral is in terms of the Förster distance, which is the distance at which energy transfer efficiency via Förster resonance energy transfer (FRET) is 50%. The efficiency of energy transfer via FRET can be generally expressed as in Equation (9) [30]. FRET is a non-radiative process through which energy is transferred from the donor (LDS material) to the acceptor (N719 dye molecules) via dipole–dipole interaction.

(9) $$\eta_{FRET}\propto {\left(\frac{R_{0}}{R}\right)}^{6}$$

where R is the distance between the donor and the acceptor molecules, and $R_{0}$ is the Förster distance at which the energy transfer efficiency is 50%. Equation (10) expresses the Förster distance in Angstrom (Å) [30].

(10) $$R_{0}=c{\left(k^{2}n^{-4}Q_{Y}J(\lambda )\right)}^{1/6}$$

$k^{2}$ is given as 2/3, which represents the relative orientation of the transition dipoles of the donors and acceptors; $Q_{Y}$ is the quantum yield of the donor in the absence of the acceptor—in this case a value of 0.27; n is the refractive index of the medium containing the donor and acceptor molecules, which is 1.33; J(λ) is the overlap integral between the donor and acceptor molecules; c is a numerical coefficient of value 0.211$M^{1/6}{cm}^{1/6}{nm}^{1/3}$. Table 1 shows the computed FRET parameters.

## 3. Results and Discussion

### 3.1. SEM Analysis

**Figure 2:**
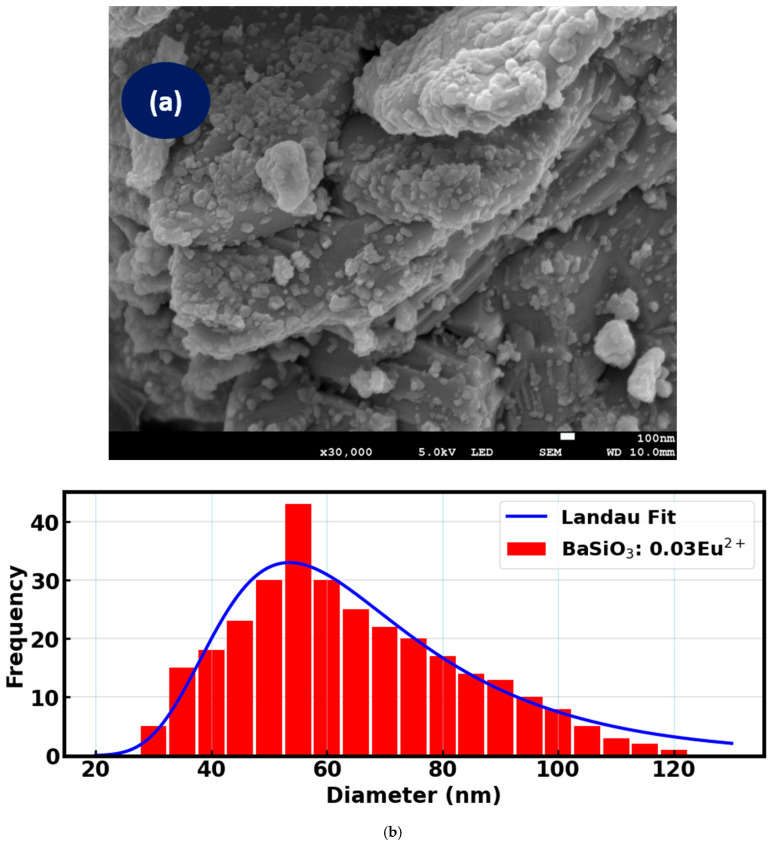
(**a**). SEM image of BaSiO_3_:0.03Eu^2+^ phosphor. (**b**). Diameter distribution of BaSiO_3_:Eu^2+^ nanoparticles.

SEM is essential for morphological analysis of the material. As depicted in Figure 2a, the surface morphology of the material shows that the sample has particle sizes of non-uniform accumulation. The surface of the sample shows irregular shapes, and this means the distribution of the particle sizes is rough and non-homogenous. The sample presents a layered and slightly flaky structure with substantial surface roughness due to the presence of rough distributed nanoparticles. The primary phase of barium orthosilicate naturally tends to form plate-like or granular morphologies based on the solid-state technique applied during synthesis of the material.

A rough and highly faceted surface morphology is advantageous for enhancing the overall phosphor emission intensity, which is beneficial for DSSCs applications [31,32]. It significantly increases the available surface area for dye molecule adsorption. Further, such a morphology promotes enhanced light scattering due to microstructural surface roughness, which improves photon absorption by redirecting incident light within the photo-anode layer. These properties suggest that the barium orthosilicate phosphor can serve effectively as a light management material in DSSC designs.

The particle size distribution of barium orthosilicate nanoparticles, centered on a mean diameter of around 54.6 nm, as shown in Figure 2b, presents several advantages for DSSCs applications. The histogram was analyzed using a Landau distribution function because it is more suitable to account for the asymmetry and positive skew effects observed in the nanoparticle size distribution. It illustrates a relatively narrow and unimodal distribution, with the majority of particles falling within the 40–80 nm range and a peak frequency in the 50–60 nm interval. The slight positive skew in the distribution, with a small number of particles exceeding 100 nm, may further assist in scattering light efficiently, as such observed particle size and roughness could promote photon absorption. This well-controlled size distribution is indicative of a consistent synthesis process. Nanoparticles with mean diameter around 54.6 nm offer a good balance between surface area and optical functionality. Smaller particles provide a high surface area for dye adsorption, which is essential for maximizing light-harvesting efficiency [33,34]. Therefore, while some larger particles are present, their lower frequency distribution suggests they are unlikely to significantly hinder charge transport.

### 3.2. EDS Analysis

**Figure 3:**
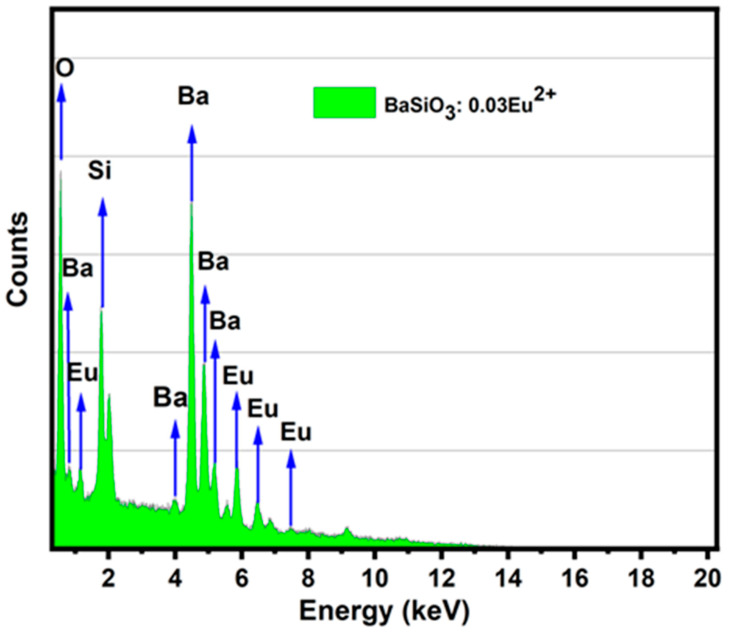
EDS spectrum of the BaSiO_3_:Eu^2+^.

The energy-dispersive X-ray spectroscopy (EDS) spectrum of the barium orthosilicate sample provides valuable insights into the elemental composition and verifies the effective synthesis of the intended doped phosphor material. As shown in Figure 3, the prominent peaks in the spectrum correspond to the barium (Ba), silicon (Si), oxygen (O), and europium (Eu), all of which are anticipated elements in the compound. Multiple strong peaks attributed to barium are observed at approximately 0.7 keV, 4 keV, 4.5 keV, 5.2 keV, and 5.7 keV, which align with the Ba Lα and Lβ emission lines. This shows that barium is a major constituent, as expected from the BaSiO_3_ matrix. A peak for silicon is visible around 1.75 keV, consistent with the presence of silicon from the orthosilicate (SiO_4_) structure. Additionally, a distinct peak at approximately 0.5 keV confirms the presence of oxygen, which is a crucial constituent of the oxide network. Europium was also detected through several small peaks ranging between 1.1 keV and 7.5 keV. These are characteristic of Eu M and L emission lines. Although Eu was present in a low concentration (3 mol%), the sensitivity of EDS enables its detection, indicating that the doping process was effective. The presence of europium peaks confirms that Eu^2+^ ions were effectively integrated into the host lattice, thereby substituting for Ba^2+^ due to their similar ionic radii.

The elemental composition supports the formation of a pure and stable BaSiO_3_:Eu^2+^ matrix. The BaSiO_3_:Eu^2+^ phosphors are widely reported in the literature [35,36] to possess good thermal and chemical stability, suggesting long-term structural reliability. As a wide bandgap material, the host can act as an efficient light-scattering layer when deposited over traditional photoanodes like TiO_2_. Meanwhile, the incorporation of Eu^2+^ brings photoluminescent properties into play. Eu^2+^ ions are known to absorb UV light and re-emit it in the visible spectrum [37,38], making the phosphor a down-shifting material for DSSCs applications. This approach leverages the energy transfer mechanism from the BaSiO_3_:Eu^2+^ donor to dye molecules, where absorbed UV photons are converted into visible emissions, which can be absorbed more efficiently by the dye to enhance spectral utilization and reduce UV-induced degradation.

### 3.3. Optical Analysis

**Figure 4:**
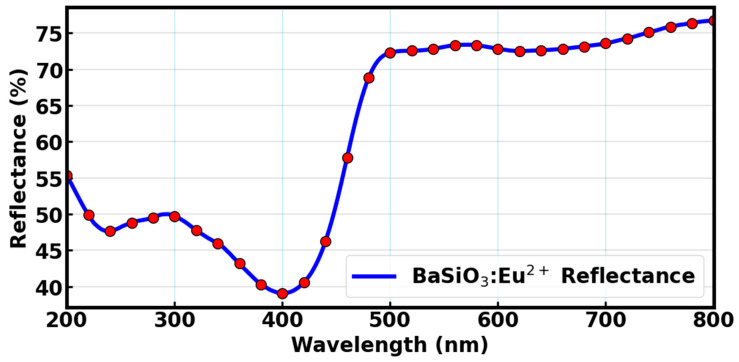
Reflectance spectrum of BaSiO_3_:Eu^2+^.

The diffused reflectance spectrum of BaSiO_3_:Eu^2+^ is presented in Figure 4. The sample shows high reflectivity in the range 410–800 nm and low reflectivity below 400 nm. This shows that within the visible and near infrared ranges, the reflectivity of the sample was high but dropped significantly in the UV region. The decrease in the reflectance spectrum in UV region can be attributed to the luminescent down-shifting properties of the BaSiO_3_:Eu^2+^ material in absorbing photons of short wavelength, typically within the UV region, and re-emitting them within the visible region due to the 4f → 5d dipole allowing electronic transition of the Eu^2+^ ions. The BaSiO_3_:Eu^2+^, being a good absorber of radiant UV photons, can be applied as a UV light-harvesting material to capture and convert the unused UV radiation in optoelectronic devices such as dye-sensitized solar cells [39,40,41]. This enhances the power output and overall performance of the device. Moreover, there is a high reflectivity of barium orthosilicate doped with europium (II) in the visible region of the solar spectrum, while the typical dyes such as N719 and organic dyes have strong absorption in the visible region. Therefore, due to its high reflectance in the visible region, BaSiO_3_:Eu^2+^ is not intended to act as a standalone top coating but can be more effectively utilized as an embedded or interfacial luminescent layer that selectively absorbs UV without interfering with visible light transmission. Also, BaSiO_3_:Eu^2+^ may not be applied as a replacement of the dye molecules but can serve as a co-sensitizer to the dyes in order to extend the absorption profile to the UV region and promote the generation of charge carriers and electron transport to the external circuit.

**Figure 5:**
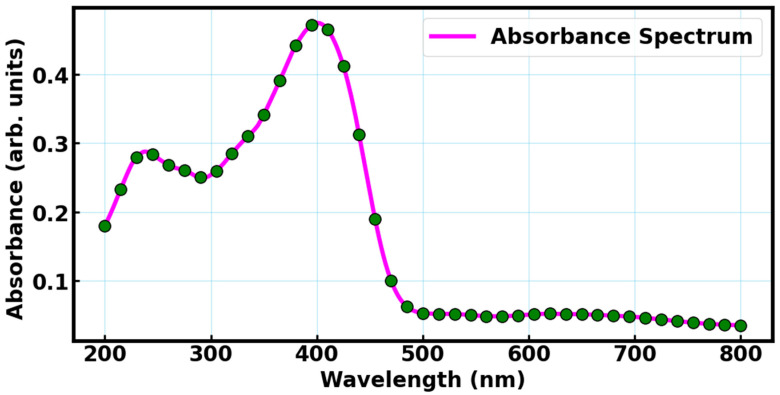
Absorption profile of BaSiO_3_:Eu^2+^ material.

Barium orthosilicate material, as demonstrated by its absorbance spectrum, exhibits strong UV absorption with a prominent peak around 400 nm and a secondary peak between 200–300 nm, as shown in Figure 5. This makes it well-suited for application as a UV-absorbing down-shifting material in DSSCs. The core function of a down-shifting material is to absorb high-energy UV photons and re-emit them at lower-energy visible wavelengths via energy transfer mechanism [42,43]. In this case, the Eu^2+^ ions doped into the BaSiO_3_ host enable this conversion via efficient 5d → 4f electronic dipole allowed transitions, producing visible emission that can be more effectively utilized by the dye molecules in a DSSC.

In DSSCs, UV light is not efficiently absorbed by conventional dye, and prolonged UV exposure can lead to degradation of both dyes and electrolytes, reducing device longevity. Incorporating a down-shifting layer such as BaSiO_3_:Eu^2+^ can address this limitation. This is via an energy transfer mechanism by absorbing harmful UV radiation and re-emitting it in the visible range. The approach not only enhances the spectral response of the device but also improves its stability. Also, the silicate host matrix is chemically and thermally stable [36,44], which is crucial for maintaining long-term performance under continuous solar exposure. Further, the down-shifting mechanism does not interfere with the internal photo-electrochemical processes of the DSSC due to strong overlap between BaSiO_3_:Eu^2+^ and the dye molecules in the DSSCs, allowing for seamless integration. This active approach to enhancing efficiency and protecting the cell materials makes BaSiO_3_:Eu^2+^ a practical and effective addition.

**Figure 6:**
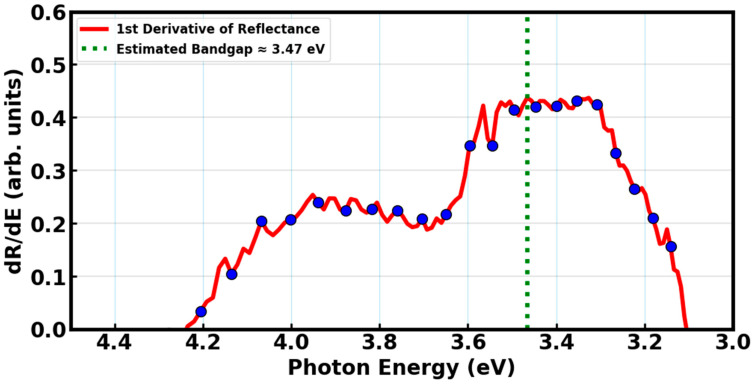
Band gap estimation of BaSiO_3_:Eu^2+^ using first derivative of reflectance.

The band gap estimation using the first derivative of diffused reflectance is shown in Figure 6. The maximum marks the limit or peak of a material, which occurs at a wavelength where the energy of the absorbed photon corresponds to the electronic transition from the valence band to the conduction band. A point at the abscissa where the peak intersects with the photon energy axis yields the bandgap of the material. In this case, the absorption edge appears to intersect around 3.47 eV, which is a reasonable estimate for the direct band gap of BaSiO_3_:Eu^2+^ material, and positions this material in the UV region of the electromagnetic spectrum [45]. For DSSCs, this band gap indicates that BaSiO_3_:Eu^2+^ material is not suitable as a primary light absorber or photo-anode material, as it does not effectively absorb visible light where the solar spectrum is most intense. However, this band gap makes the material very effective at absorbing UV radiation, aligning with its potential application as a down-shifting material.

As discussed in conjunction with the absorbance and reflectance spectra, BaSiO_3_:Eu^2+^ can absorb UV photons and re-emit them as visible light through the characteristics 5d → 4f electronic transitions of Eu^2+^ ions. Via the energy transfer mechanism, the emitted visible light can then be absorbed by the dye molecules in DSSCs such as ruthenium N719 or organic dyes, which are most effective in the 400–700 nm range [46,47,48]. Therefore, the 3.47 eV band gap reinforces the role of BaSiO_3_:Eu^2+^ as a spectral converter that enhances DSSC performance indirectly. It protects the cell from UV degradation and boosts performance by converting unused UV light into usable visible light.

### 3.4. Photoluminescent Analysis

Photoluminescence is the emission of light from a material upon excitation by photons, and its spectra can provide insights into energy-level transitions within the material. A material under photo-excitation would have its electrons moved to the allowed excited states. The electrons are in the process of returning to the ground state emit energy, which may include the emission of light (luminescent/radiative process) or may not (non-radiative process). While recording the photoluminescence, both the excitation and emission spectra were scanned. A variation of the signal/intensity with wavelength, time, or other variables may be observed in the system, depending on the nature of the variable.

**Figure 7:**
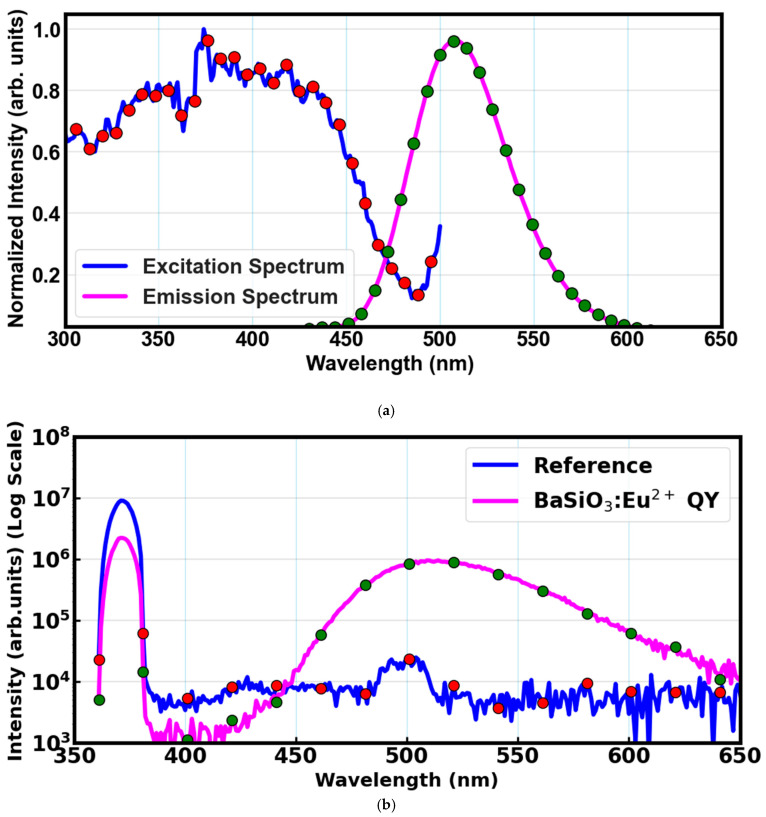
(**a**) Photoluminescence excitation and emission spectra of BaSiO_3_:Eu^2+^ material. (**b**) Quantum yield of BaSiO_3_:Eu^2+^ material with the reference.

The photoluminescence excitation (PLE) and emission (PL) spectra of the phosphor material are shown in Figure 7a. The excitation spectrum was recorded by monitoring emission at 511 nm, revealing a broad absorption band peaking at 371 nm, while the emission spectrum under 371 nm excitation shows a prominent emission centered at 511 nm. The PLE spectrum presents a broad absorption band from approximately 300–460 nm and centered at 371 nm. This can be attributed to the electronic dipole allowed 4f^7^ → 4f^6^5d^1^ transition of BaSiO_3_: Eu^2+^ ions [49,50]. Broad emission centered at around 511 nm corresponds to the 4f^6^5d^1^ → 4f^7^ transition, which is a characteristic of Eu^2+^ ions. The Eu^3+^ would instead show sharp line emissions due to f → f transitions, which in this case are not represented in our spectra. The Stokes shift (∆λ) was calculated to be greater than 100 nm from the peak of the excitation spectrum (excitation wavelength: 371 nm) to the peak of the emission spectrum (emission wavelength: 511 nm). This indicates a broad and strong band spectrum in the visible region, showing there is less chance of the emitted light being re-absorbed by the material itself, which helps reduce energy loss. Since DSSCs do not efficiently absorb the UV light, material like BaSiO_3_:Eu^2+^ can effectively capture the UV/blue light and re-emit it as green light—which can then be absorbed by the dye molecules of DSSCs. The photoluminescent analysis positioned BaSiO_3_:Eu^2+^ as a UV-absorbing down-shifting material, suitable for DSSC applications. Through the mechanism of energy transfer, the absorbed UV photons are re-emitted to the visible wavelengths for better utilization in DSSC devices.

Figure 7b shows the quantum yield of the BaSiO_3_:Eu^2+^ with the reference. Emission spectra of both the reference and the sample were measured under the same 371 nm excitation, and the ratio of the integrated emission with respect to the integrated excitation was used to determine the quantum yield of the material. The fluorescence emission spectrum of the material and the excitation spectrum of the reference were collected using an integrating sphere. The quantum yield of the BaSiO_3_:Eu^2+^ was determined to be 0.27. This value was used to estimate the spectral overlap integral via Förster resonance energy transfer (FRET).

**Figure 8:**
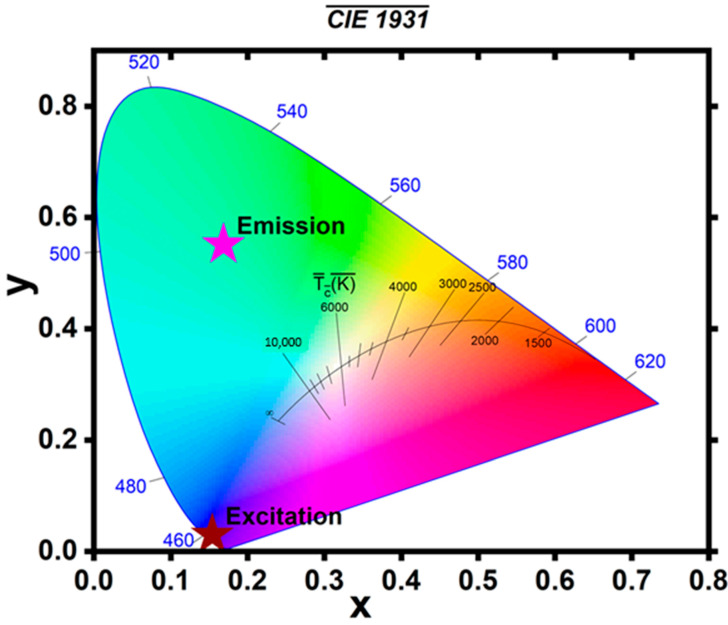
CIE 1931 chromaticity diagram of the BaSiO_3_:Eu^2+^ for both excitation and emission wavelengths.

The CIE (Commission Internationale de l’Éclairage—International Commission on Illumination) 1931 chromaticity diagram, as shown in Figure 8, was used to identify the excitation and emission color of the barium orthosilicate doped with europium (II) from the photoluminescent emission spectra. The diagram gives an understanding of how the material interacts with light in terms of color, for example, what kind of light it absorbs and emits. The excitation color of the BaSiO_3_:Eu^2+^ was deep blue violet at the corner of the (x = 0.15405, y = 0.02969) coordinate, while the emission color was found to be within the green region at the corner of the (x = 0.16875, y = 0.5513) coordinate, showing that after absorbing energy, the material emitted green-colored light. In this context, the CIE 1931 diagram was used primarily to visualize the wavelength shift and qualitatively illustrates the shift from UV excitation to visible green emission. It can be seen from the CIE diagram that the BaSiO_3_:Eu^2+^ has shifted its wavelength from the blue UV region to the visible green region of the diagram, thereby demonstrating the luminescent down-shifting ability of the material in absorbing photons of short wavelengths within the UV and re-emitting them at longer wavelength of the visible region. When integrated into the DSSCs, the material could extend the absorption spectrum of the devices to UV to capture and convert incidence irradiation into usable energy [51,52], hence enhancing the light-harvesting capacity of the devices to mitigate recombination losses and promote generation of charge carriers, which could improve the power conversion efficiency of DSSCs.

### 3.5. Energy Transfer Mechanism

**Figure 9:**
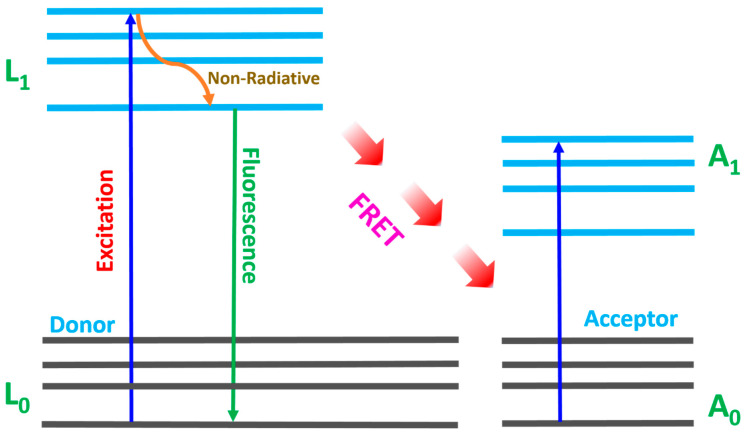
Förster Resonance Energy Transfer Mechanism.

Energy transfer from the luminescent material to the N719 dye molecules primarily occurs via Förster resonance energy transfer (FRET). FRET is a process where energy is transferred from the donor (LDS material) to the acceptor (dye molecules), mediated by the dipole–dipole interactions [53,54,55]. The mechanism is very much dependent on the distance between the donor and the acceptor, usually occurring over the range of 1–10 nm. Figure 9 shows the mechanism by which the donor electrons transition from the ground state (L0) to the excited state (L1). Non-radiative transition occurs within the lower level of the excited state, which could be attributed to the phonon. Thereafter, emission and energy transfer to the acceptor molecules via FRET mediation. The excited molecules transition from the excited state to the ground state, which necessitates continuous energy transfer.

**Figure 10:**
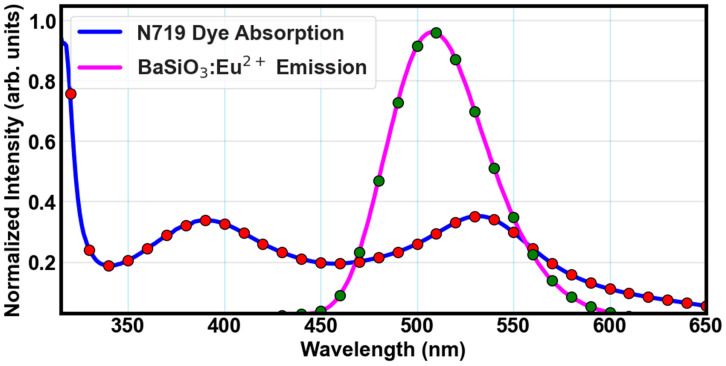
Spectral overlap of the N719 dye acceptor and BaSiO_3_:Eu^2+^ donor.

The distance between the donor and the acceptor molecules as well as the strength of the overlap integral determines the efficiency of energy transfer. In other words, the smaller the distance between the donor and the acceptor molecules and the larger the strength of the overlap integral, the greater the efficiency of the energy transfer. FRET spectral integral between the emission spectrum of BaSiO_3_:Eu^2+^ and the absorption spectrum of the N719 dye molecules is depicted in Figure 10. A strong overlap was achieved between the phosphor material and the dye molecules, and an estimated spectral overlap integral J(λ) value of $2\times 10^{10}M^{-1}{cm}^{-1}{nm}^{4}$ was realized. This value is large enough, indicating an efficient energy transfer process [29,55]. J(λ) was computed by integrating the emission spectrum of the donor and the absorption spectrum of the acceptor and weighted to the fourth power of the wavelength, as depicted in Equation (4). The integration was performed numerically in Python 3.9 using the Simpson’s rule across the spectral overlap region. The high J(λ) value was due to the significant overlap in the approximate 460–560 nm range shown in Figure 10. The BaSiO_3_:Eu^2+^ material facilitated the energy transfer mechanism, which emphasizes the importance of the luminescent down-shifting material in the development of efficient energy transfer systems. The computed FRET parameters are shown in Table 1.

Moreover, the N719 dye could not absorb the UV radiation in the range around 200–350 nm, and the UV absorption in this region dwindled evanescently, which could cause thermal losses of charge carriers by high-energy photons in the region and could be detrimental to the overall DSSC performance due to degradation of the dye molecules by prolonged high-energy UV exposure. The BaSiO_3_:Eu^2+^ material, being an efficient UV absorber, is capable of absorbing photons within this UV region and re-emitting them to the visible wavelength range, where N719 dye molecules can easily absorb them to facilitate even more generation of charge carriers. However, the LDS material not only enhances the absorption of the unused UV photons but also renders protection of the DSSC device against harmful UV exposure.

**Figure 11:**
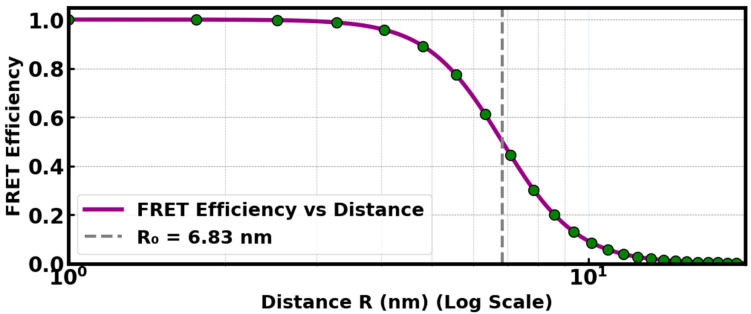
FRET efficiency as a function of distance between the donor and acceptor molecules.

FRET efficiency as a function of distance between the donor and the acceptor molecules is depicted as shown in Figure 11 below. It can be deduced that the FRET efficiency decreases as the distance between the donor and the acceptor increases, which demonstrates that FRET is always effective at a typically close distance. $R_{0}=6.83nm$ indicates the participation of FRET mechanism, which falls within the range 1–10 nm. Therefore, at a distance of 6.83 nm, the energy transfer between the donor, BaSiO_3_:Eu^2+^, and the acceptor, the N719 dye, would be 50% efficient. Estimation of $R_{0}$ was computed from the relationship governing the Förster distance, the quantum yield of the donor in the absence of the acceptor, the spectra overlap between the donor and acceptor molecules, the refractive index of the medium containing the donor and the acceptor molecules, and the relative orientation of the transition dipoles of the donor and acceptor—depicted as shown in Equation (6).

Generally, the development of an efficient energy transfer system is strongly dependent on the Förster distance between the donor and acceptor molecules as well as the strength of the spectra overlap integral between the donor and acceptor. A system with a Förster distance below 10 nm and a strong overlap between the donor and the acceptor molecules would produce efficient energy transfer. This mechanism in DSSC would facilitate generation of charge carriers that would promote the power output and the overall performance of the device.

## 4. Conclusions

This research presents the synthesis and optical, photoluminescent, and energy transfer analysis of barium orthosilicate doped with europium (II) (BaSiO_3_:Eu^2+^) as a luminescence down-shifting (LDS) material for dye-sensitized solar cells (DSSCs). The material’s strong ultraviolet (UV) absorption and broad visible emission indicate its suitability in converting high-energy UV photons into lower-energy ones compatible with typical DSSC sensitizers such as N719. In this study, the material was synthesized via conventional solid-state technique. Scanning electron microscope (SEM) analysis revealed non-uniform particle distribution advantageous for enhanced light scattering in the DSSCs. Energy-dispersive X-ray spectroscopy (EDS) provided valuable insight into the elemental composition of synthesized material, which confirms the crystal formation of BaSiO_3_:Eu^2+^.

Photoluminescent analysis depicted a strong broadband absorption in the UV region, and the Stokes shift was calculated to be greater than 100 nm, which indicates strong emission in the visible region of the solar spectrum, suggesting reduced self-absorption losses. Quantitative analysis based on Förster resonance energy transfer (FRET) yielded a Förster distance of 6.83 nm. A strong overlap was realized between the BaSiO_3_:Eu^2+^ donor and the N719 dye acceptor, and the spectral overlap integral was estimated to be $2\times 10^{10}M^{-1}{cm}^{-1}{nm}^{4}$, which is significant for facilitating efficient energy transfer. These attributes highlight BaSiO_3_:Eu^2+^ as a viable LDS material that can improve DSSC performance through extending the absorption profile of DSSC to the UV region to facilitate enhanced device performance.

The material’s optimization and integration into photovoltaic devices could play a vital role in meeting the global energy demands and environmental goals, proving beneficial for generations to come. Although this research presented the spectral compatibility and energy transfer potential of BaSiO_3_:Eu^2+^ with N719 dyes, experimental integration with actual devices under operational conditions remains crucial, and we clearly acknowledge this limitation. However, this will be the focus of our future studies.

## Figures and Tables

**Table 1:** FRET parameters.

$c(M^{1/6}{cm}^{1/6}{nm}^{1/3})$	$k^{2}$	$Q_{Y}$	n	$J(\lambda )(M^{-1}{cm}^{-1}{nm}^{4}$ **)**	$R_{0}$ (nm)
0.211	2/3	0.27	1.33	$2\times 10^{10}$	6.83

## Data Availability

The original contributions presented in this study are included in the article. Further inquiries can be directed to the corresponding author.

## Abbreviations

The following abbreviations are used in this manuscript:

EDS	Energy-dispersive X-ray spectroscopy
DSSCs	Dye-sensitized solar cells
FRET	Förster resonance energy transfer
LDS	Luminescent down-shifting
SEM	Scanning electron microscope
UV	Ultraviolet
